# Supplementary material for: Voltage-gated sodium channels assemble and gate as dimers
Source: Nat Commun. 2017 Dec 12;8:2077. doi: 10.1038/s41467-017-02262-0 (PMC5727259; doi:10.1038/s41467-017-02262-0)
Supplement: Supplementary file 1 — Supplementary Information [file 41467_2017_2262_MOESM1_ESM.pdf]

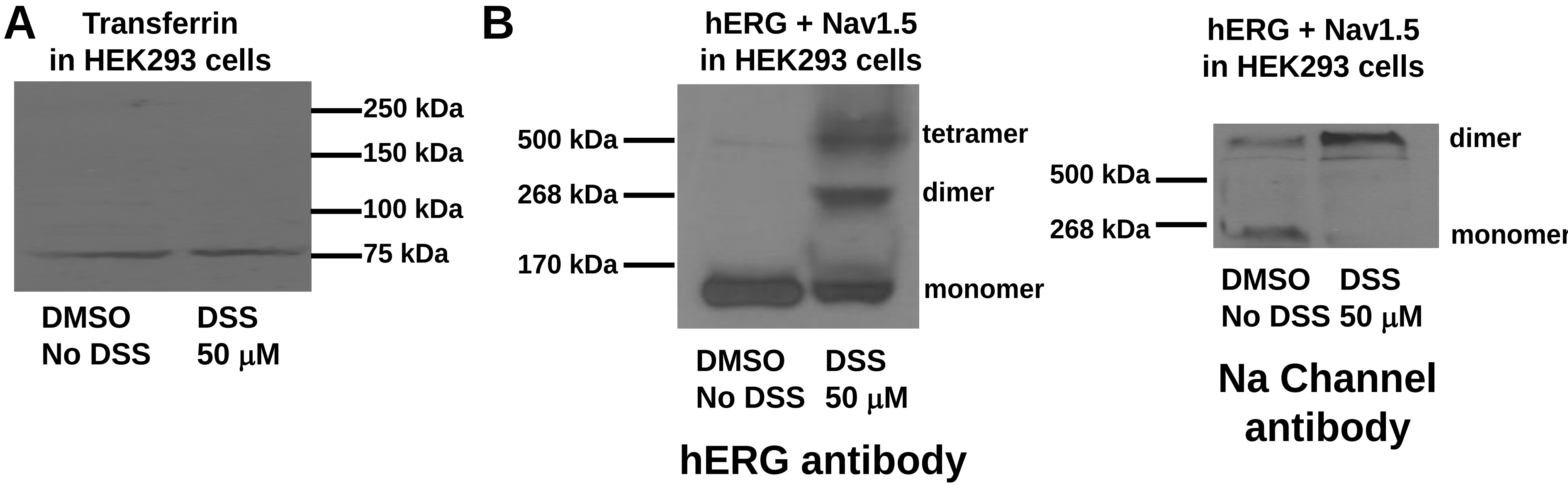

Supplementary Figure 1: Control for Crosslinking Experiments. **A.** Crosslinking experiments performed with DSS in HEK293 cells expressing transferrin. Crosslinking was performed using DSS at 50 μM for 20 minutes. Only a monomeric band was observed for transferrin. **B.** Crosslinking experiments performed with DSS in HEK293 cells co-transfected with hERG and Na_v1.5. Crosslinking was performed using DSS at 50 μM for 20 minutes. Bands corresponding to a monomer, dimer and tetramer were observed for hERG in presence of the crosslinker compared to cells with no DSS. Bands corresponding to a monomer and dimer of Na_v1.5 α-subunit is observed in presence of the crosslinker compared to the cells where no crosslinker was present (DMSO no DSS). Full blots are presented in Supplementary Figure 14.

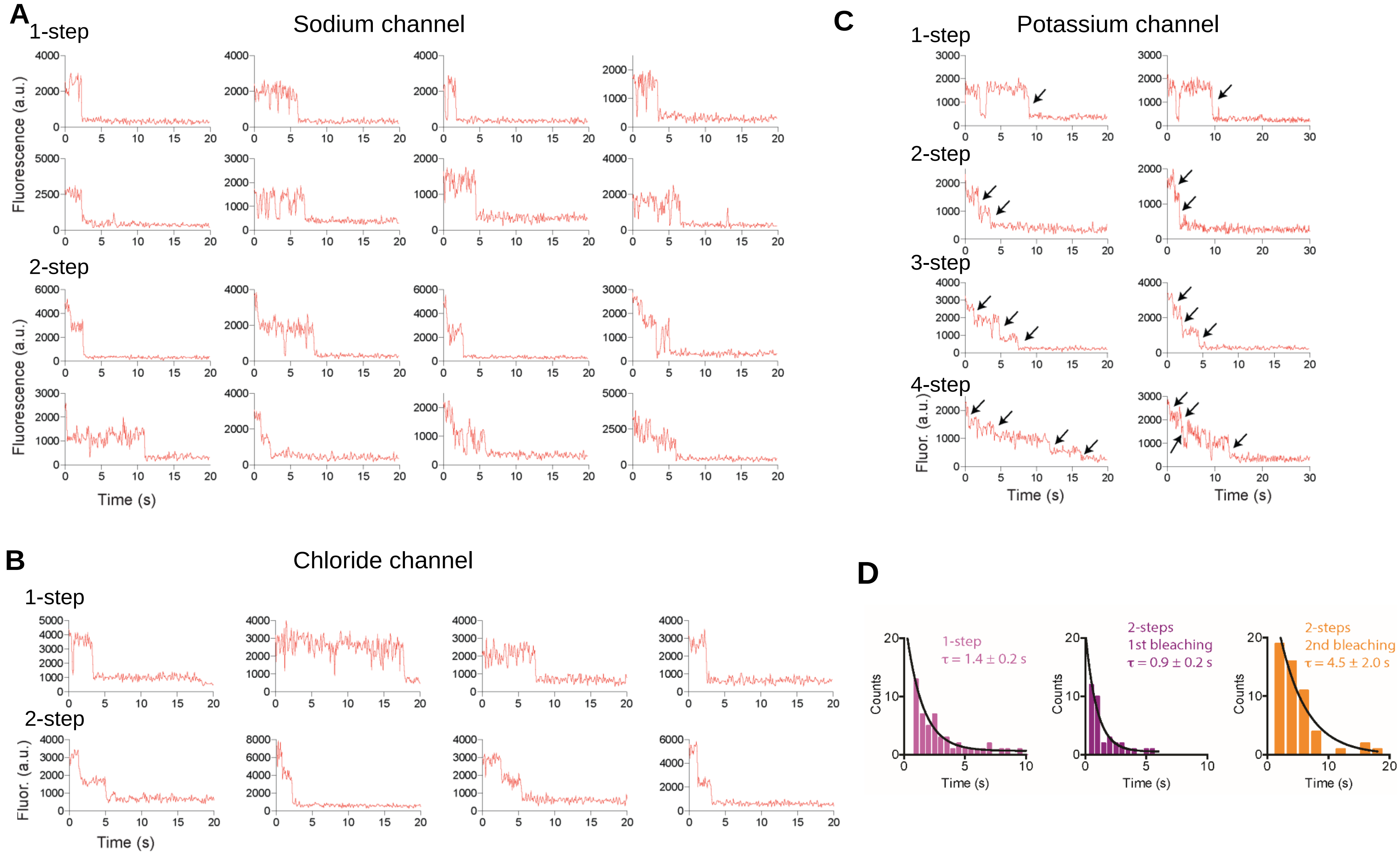

Supplementary Figure 2: Representative SiMPull photobleaching step traces for **A.** Sodium Channel Na$_v$1.5 **B.** Chloride Channel ClC-3 **C.** Potassium Channel Kv4.3. Y axis represents the fluorescence intensity and the X axis is time. In panel A for the sodium channel, examples of 1 (first two rows) and 2 (lower two rows) GFP photobleaching steps are illustrated. In panel B, for the chloride channel, examples of 1 GFP photobleaching steps are illustrated in the top row and examples of 2 photobleaching steps are illustrated in the lower row. In panel C, for the potassium channel, examples of 1, 2, 3 and 4 GFP photobleaching steps are provided. Arrows indicate the different steps. **D.** Distribution of fluorescence photobleaching times for molecules bleaching in one-step only and a corresponding fit (left). Distribution of fluorescence photobleaching times for the first (middle) and second (right) bleaching events among the molecules bleaching in two discrete steps and corresponding fits. Mean ± sem of the time constant is provided in the insets.

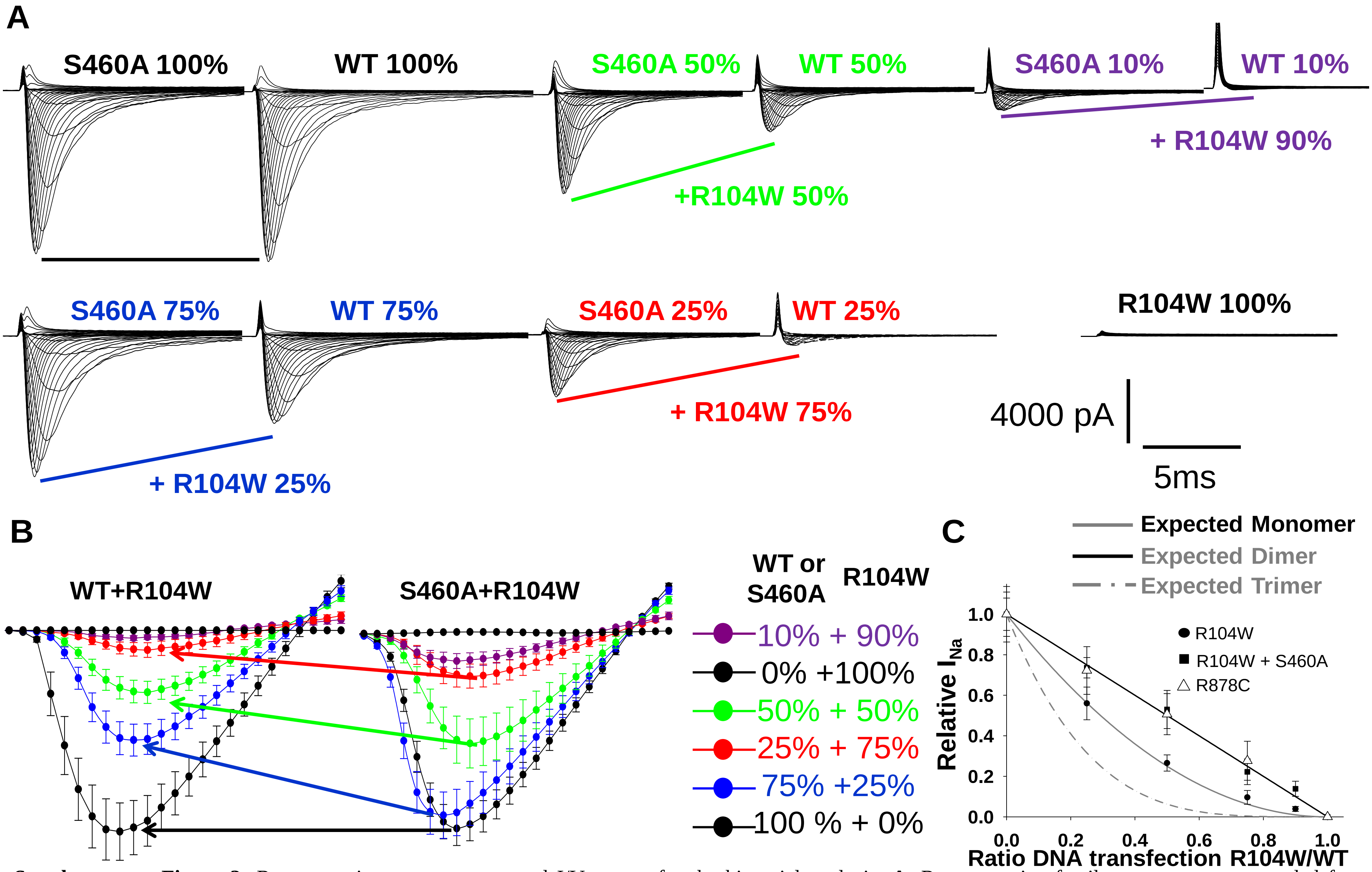

Supplementary Figure 3: Representative current traces and I/V curves for the binomial analysis. **A.** Representative family current traces recorded from HEK293 cells coexpressing WT-Na_v1.5 or S460A-Na_v1.5 with the DN-mutant R104W at the different ratios indicated by the % level of each channel. Each color corresponds to a different ratio. **B**. Corresponding I/V curves containing.. Data are presented as mean ±SEM. Arrows are used to demonstrate how the peak current for the equivalent ratio does not show a DN-effect in presence of S460A. **C.** Binomial analysis performed from transfected HEK293 cells using cDNA ratios 1:0, 10:1, 4:1 and 1:1 for WT:R104W, S460A:R104W or WT:R878C. Current densities measured at -20 mV were normalized to the 1:0 WT or S460A currents for each ratios studied. Refer to Supplementary Table 1 for n. Data points are presented as mean ± SEM.

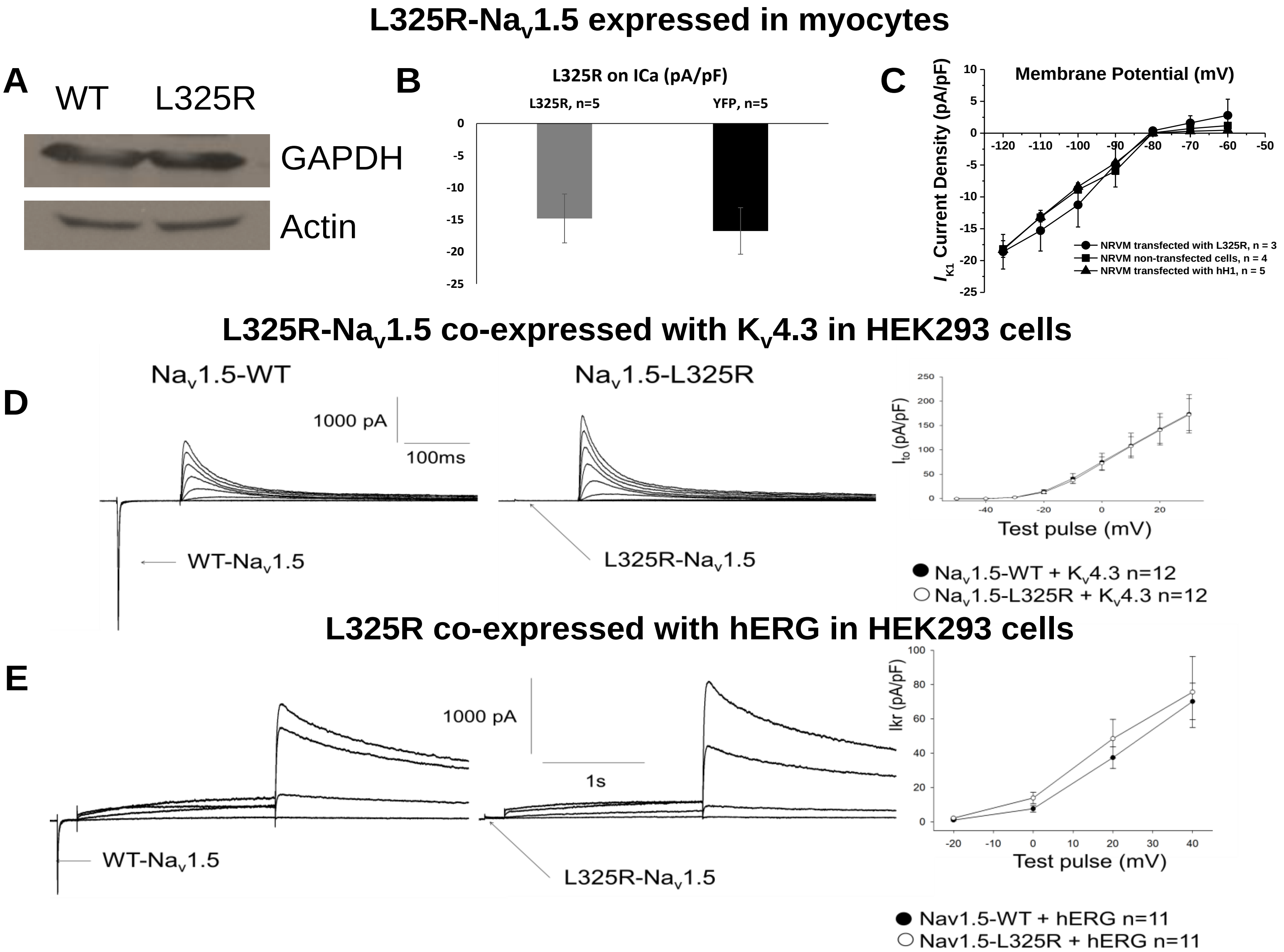

Supplementary Figure 4: Effect of L325R DN-mutant on other major cardiac currents. **A.** Western blot measuring the expression level of endogenously expressed Actin and GAPDH in presence of either the WT sodium channel or the DN-L325R mutant in HEK293 cells. Full blots are presented in Supplementary Figure 14. **B.** I_{ca} current density measured at 0mV for iCells® cardiac myocytes transfected with either YFP or the DN-mutant L325R. **C.** I_{K1} current-voltage relationship from neonatal rat ventricular myocytes transfected with either YFP or L325R. **D and E.** I_{to} and I_{Kr} current voltage relationships measured in HEK293 cells co-expressing either Na_v1.5-WT or L325R with Kv4.3 (**D**) or hERG (**E**). Representative current traces are illustrated in the left and summary I/V curves are show on the right. A short depolarizing pulse at -20mV was first made to measure and inactivate the sodium current. As can be seen with L325R no sodium current was present as expected for this mutant channel. **Note:** Importantly, no significant differences were observed on the expression of endogenous proteins as well as major currents when L325R mutant was expressed compare to WT-Na_v1.5, thus supporting that the DN-effect is specific to

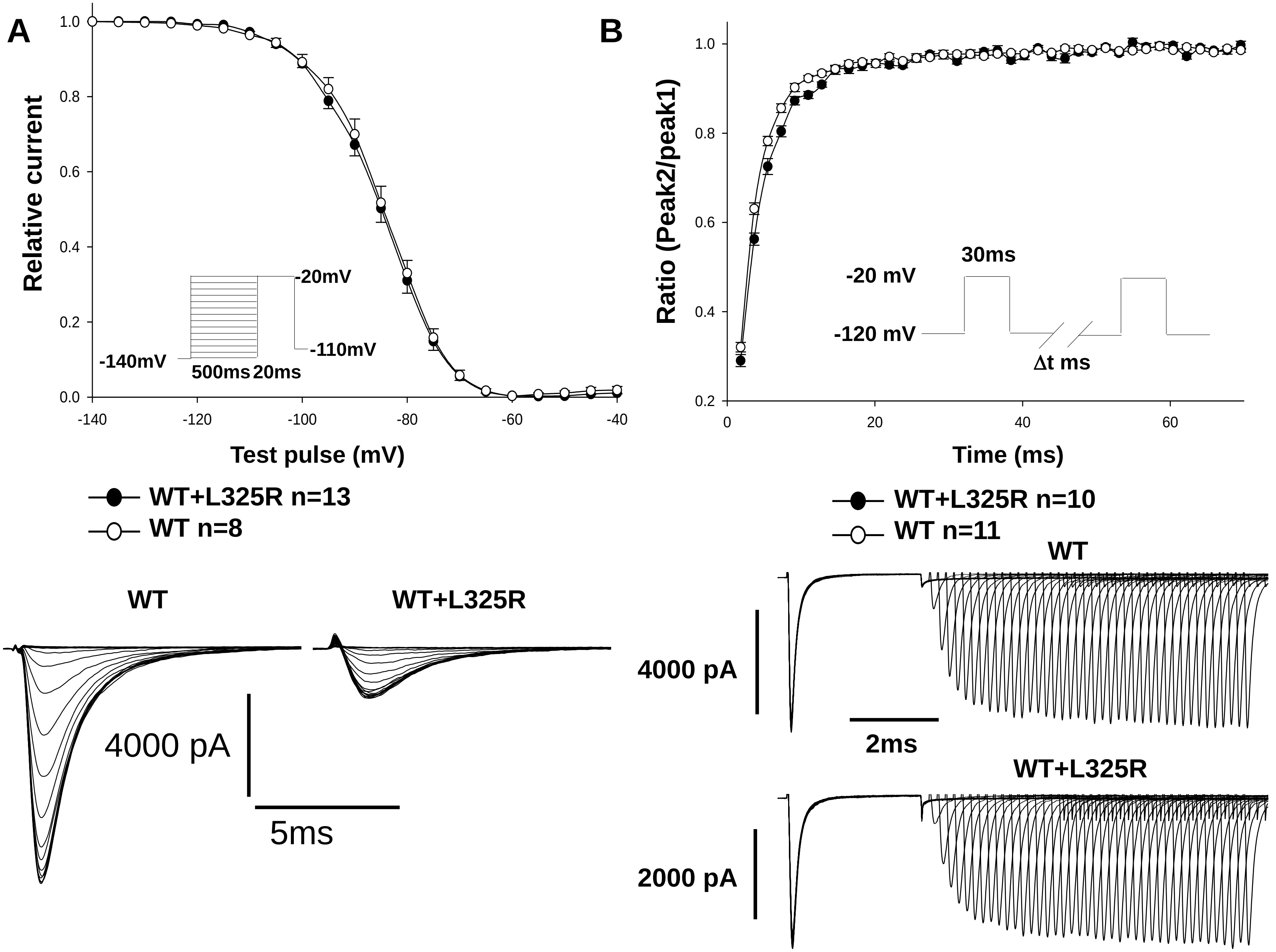

Supplementary Figure 5: No Biophysical Effect of L325R on $Na_v1.5$-WT. **A.** Steady-state inactivation and **B.** Recovery from inactivation. Top panels illustrate summary data whereas the lower panels display representative current recordings for each conditions. Data points are presented as mean ± SEM. **Note:** L325R mutant has no effect on the WT steady-state inactivation and recovery from inactivation of the WT current supporting that the DN-effect of this mutant is not caused by shifts in biophysical properties of the channel but is due to a suppressive effect mediated by the interaction of the channels.

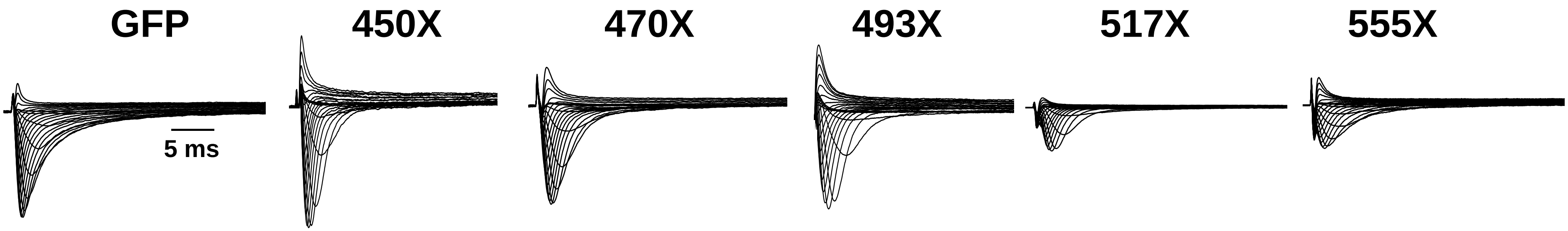

Supplementary Figure 6: Representative examples of family current traces recorded from iCells® expressing the different truncated sodium channels. Currents are normalized to GFP.

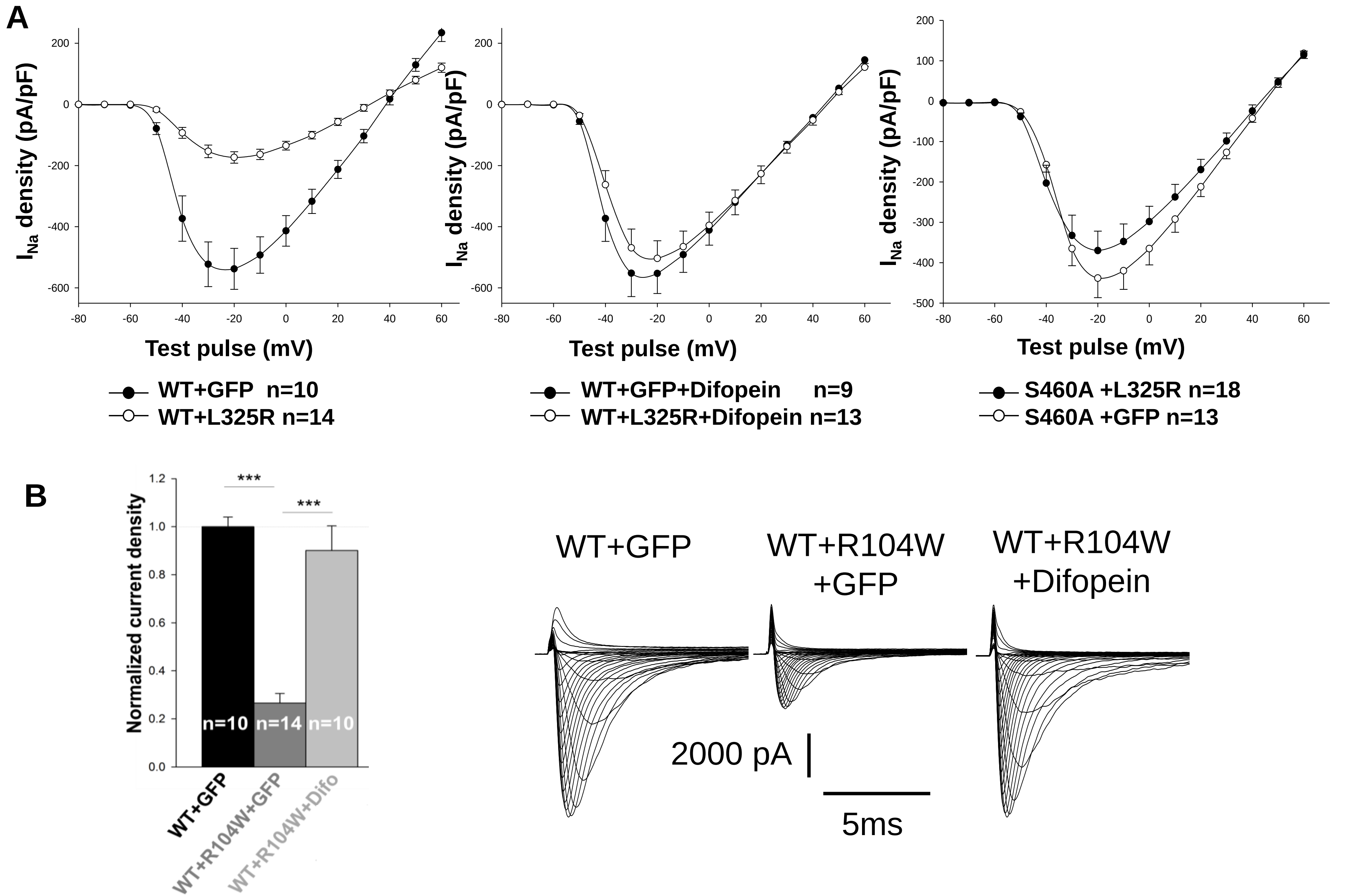

Supplementary Figure 7: 14-3-3 inhibition abolishes the DN-effect. **A.** Current voltage relationship curves recorded from HEK293 cells transfected with the conditions listed. **B.** Normalized current density at -20mV recorded from HEK293 cells coexpressing WT cardiac sodium channel with the DN-mutant R104W and GFP or the 14-3-3 inhibitor Difopein. Right panel shows representative examples of family current traces. Data points are presented as mean ± SEM.

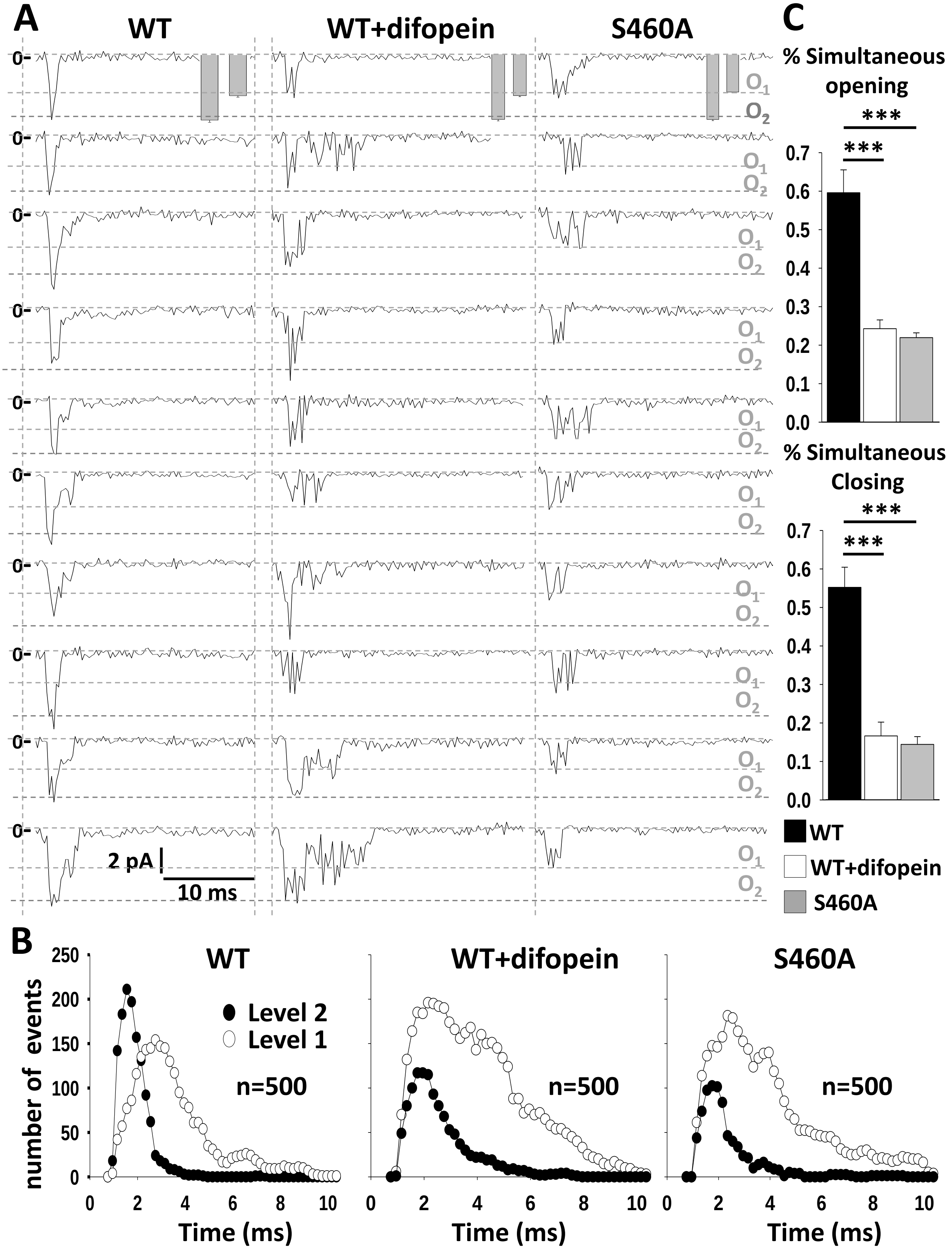

Supplementary Figure 8: $Na_v1.5$ single-channels recordings display coupled gating mediated by 14-3-3. **A**. Representative traces of WT, WT+difopein and S460A recorded at a depolarizing pulse of -20mV. The overall amplitudes for single and double openings are represented as mean ±SEM in the grey bars. The dotted lines correspond to single-level openings (O_1) and double-level openings (O_2) respectively. **B.** Number of single or double openings in function of time. Number of sweeps analyzed was 500 for each. **C.** Percentage of simultaneous openings and closings (coupled gating) observed. Data are presented as mean ±sem.

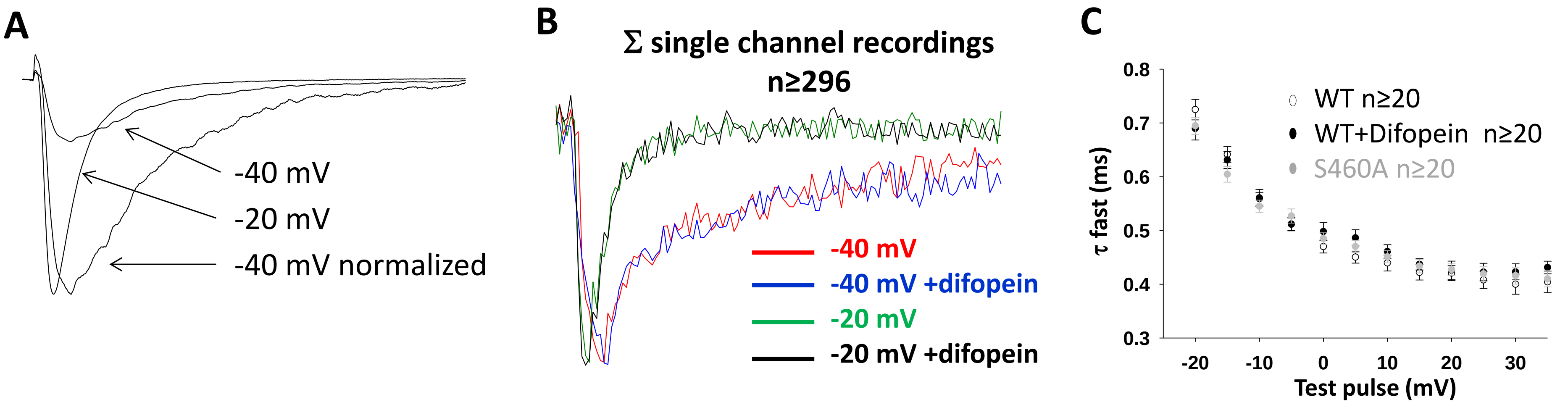

Supplementary Figure 9: A. Representative I_{Na} traces for WT channel at -20mV, -40mV, and -40mV normalized to -20mV I_{Na} peak. **B.** Sum of single-channel recordings, n≥296 sweeps at -20 and -40mV normalized with and without difopein. Overlap of the curves with and without difopein demonstrates that difopein does not affect fast inactivation of the channel even though more disperse singlelevel openings are observed. **C.** Time constants of inactivation for WT, WT+Difopein or S460A. Data points are presented as mean ± SEM.

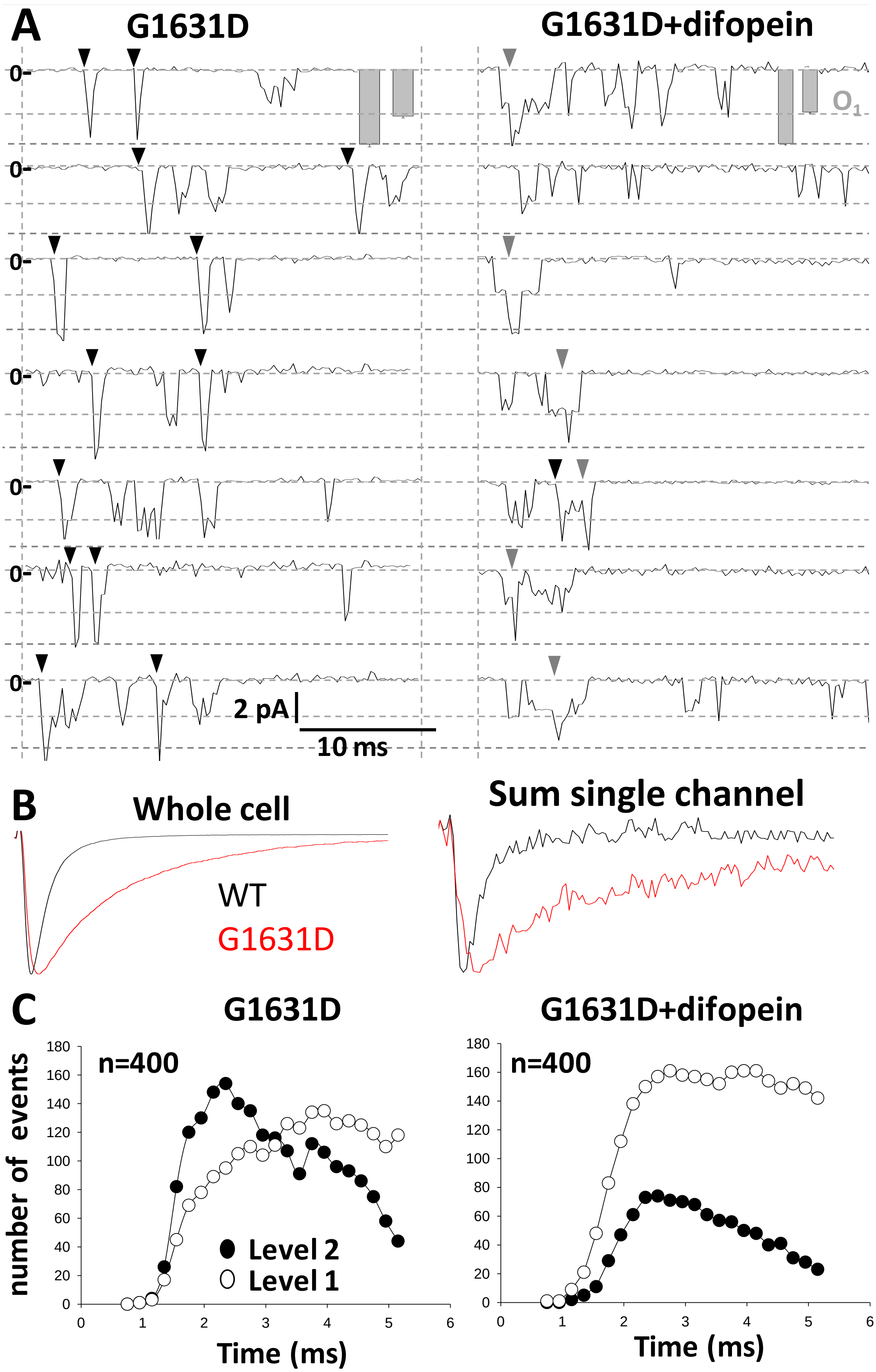

Supplementary Figure 10: Inactivation-Deficient mutant Na_v1.5-G1631D displays coupled gating mediated by 14-3-3. **A**. Representative traces of G1631D and G1631D+difopein recorded at a depolarizing pulse of -40mV. The overall amplitudes for single and double openings are represented as mean ±SEM in the grey bars. **B.** Normalized whole-cell current traces (left) for the WT (black) and G1631D (red) illustrating the inactivation defect for G163D. On the right a sum of the single-channel recordings for both WT (black) and G1631D (red) also demonstrate the inactivation defect of the mutant. **C.** Number of single or double openings in function of time. Number of sweeps analyzed was 400 for each.

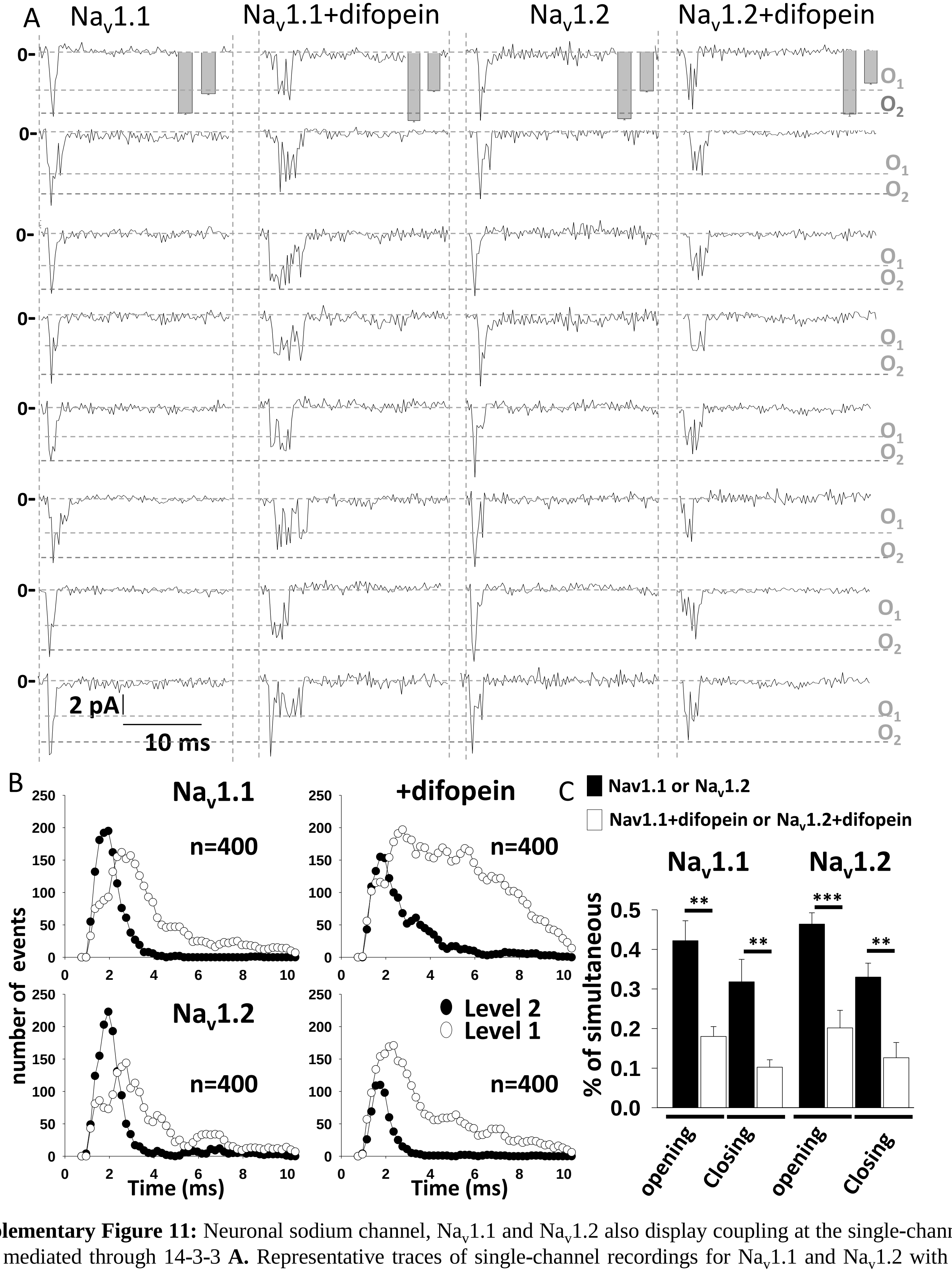

Supplementary Figure 11: Neuronal sodium channel, Na$_v$1.1 and Na$_v$1.2 also display coupling at the single-channel level mediated through 14-3-3 **A.** Representative traces of single-channel recordings for Na$_v$1.1 and Na$_v$1.2 with or without difopein at -20mV. The overall amplitudes for single and double openings are represented as mean ±SEM, grey bars. The dotted lines correspond to single-level openings (O_1) and double-level openings (O_2) respectively. **B.** The number of single openings and double openings are plotted in function of time. **C.** Percentage of simultaneous openings and closings (coupled gating) observed. Data are presented as mean ±SEM, ** p<0.01, ***p<0.001.

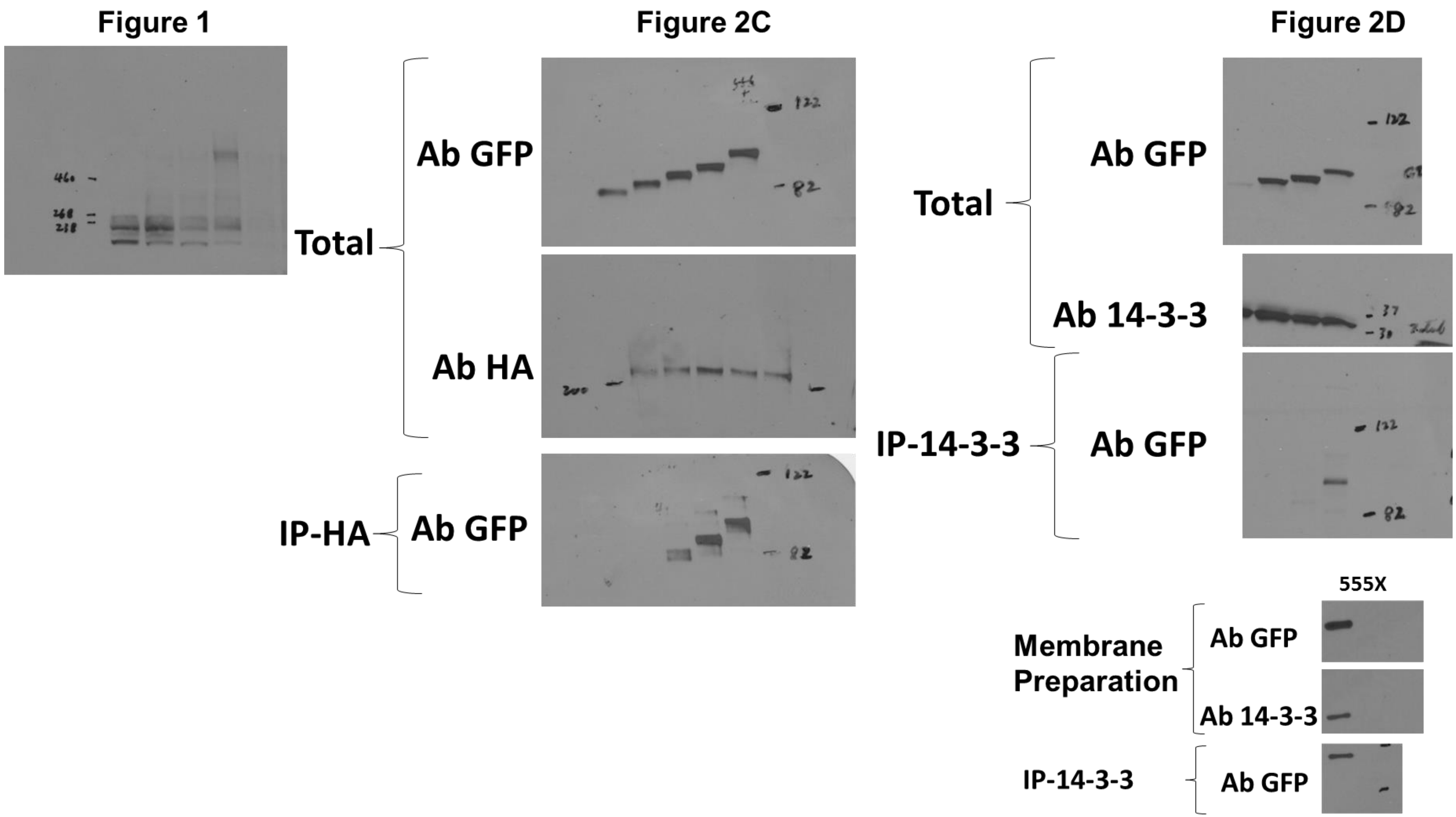

Supplementary Figure 12: Uncropped Blots for Figures 1, 2C and 2D

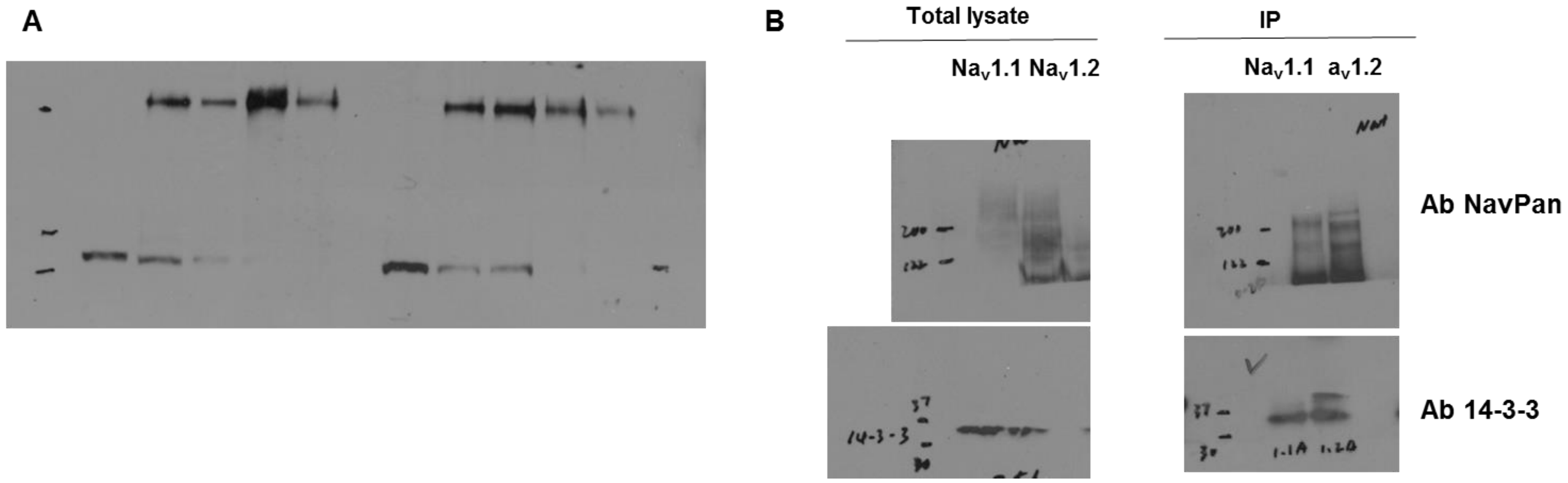

Supplementary Figure 13: Uncropped Blots for Figure 8

Suppl. Fig. 1A

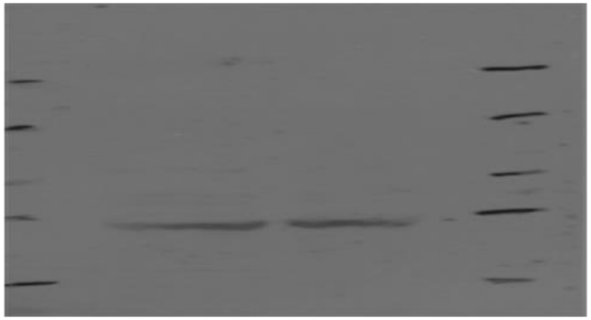

Suppl. Fig. 1B (hERG Ab)

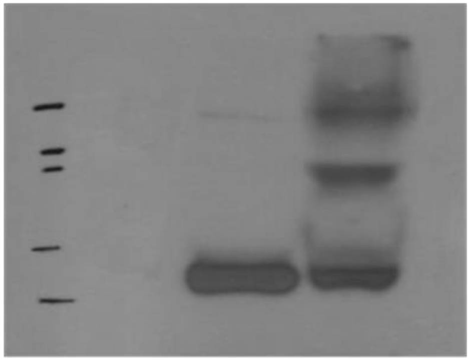

Suppl. Fig. 1B (NaCh Ab)

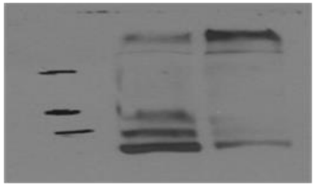

Suppl. Fig. 4A

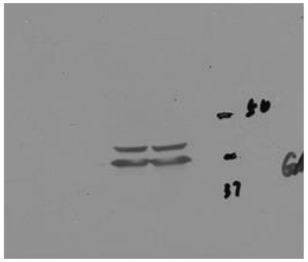

GAPDH

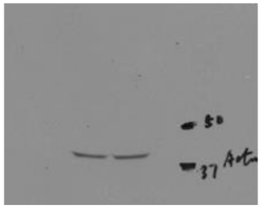

Actin

Supplementary Figure 14: Uncropped Blots for Supplementary Figures 1A-1B and Supplementary Figure 4A

Supplementary Table 1: Number of experiments (n) for the different binomial analysis from Fig. 1C and Supplementary Figure 3. ND: not done

**Mutant:WT ratio**	**0**	**0.1**	**0.25**	**0.5**	**0.75**	**0.9**	**1**
WT+L325R	62	16	33	51	49	30	12
WT+R104W	55	ND	19	39	13	13	15
WT+R878C	31	ND	17	31	19	ND	12
S460A+L325R	45	30	16	12	39	16	12
S460A+R104W	46	15	28	14	18	14	12
WT+L325R+Difopein	22	27	32	30	30	12	12
WT+R104W+Difopein	42	ND	24	15	15	25	12

Supplementary Table 2: List of all Primers Used in this Study

SCN5A L325R.F	CCTCTGATGTGTTAC**G**GTGTGGGAACAGCTCTGACG
SCN5A L325R.R	CGTCAGAGCTGTTCCCACAC**C**GTAACACATCAGAGG
SCN5A R878C.F	CTCAGGCCTGCTGCCT**T**GCTGGCACATGATGGAC
SCN5A R878C.R	GTCCATCATGTGCCAGC**A**AGGCAGCAGGCCTGAG
SCN5A S460A.F	CCGTGTCCCGTAGC**G**CCTTGGAGATGTCCCC
SCN5A S460A.R	GGGGACATCTCCAAGG**C**GCTACGGGACACGG
SCN5A G1631D.F	CCTGGCCCGAATAG**A**CCGCATCCTCAGAC
SCN5A G1631D.R	GTCTGAGGATGCGG**T**CTATTCGGGCCAGG
SCN5A R1629Q.F	CATCCGCCTGGCCC**A**AATAGGCCGCATCCTC
SCN5A R1629Q.R	GAGGATGCGGCCTATT**T**GGGCCAGGCGGATG
Fragments Xho1, F	GTCAAGATTCTGG**CTCGAG**GCTTCTGCCTG
450X EcoR1, R	CG**GAATTCtca**GATGGTGAGGGCCTCGTG
555X EcoR1, R	CG**GAATTCtca**CTCGCTCTCCCCCGCTGTG
SCN5A 450XX.F	GGCCCTCACCATC**TGATGA**AGGGGTGTGGATACC
SCN5A 450XX.R	GGTATCCACACCCCT**TCATCA**GATGGTGAGGGCC
SCN5A 555XX.F	GGGGAGAGCGAG**TGATGA**AGCCACCACACATCAC
SCN5A 555XX.R	GTGATGTGTGGTGGCT**TCATCA**CTCGCTCTCCCC
SCN5A 517X.F	CGTGGCCTCAGCAGG**TGA**ACTTCTATGAAGCCAC
SCN5A 517X.R	GTGGCTTCATAGAAGT**TCA**CCTGCTGAGGCCACG
SCN5A 493X.F	GTGTGGGGAGGACAGG**TGA**CTCCCCAAGTCTGAC
SCN5A 493X.R	GTCAGACTTGGGGAG**TCA**CCTGTCCTCCCCACAC
SCN5A 470X.F	GGCCCCAGTAAACTGAAGCCA**TGA**GAGAAGAAGC
SCN5A 470X.R	GCTTCTTCTC**TCA**TGGCTTGAGTTTACTGGGGCC
